# Differentiating placenta accreta spectrum from scar dehiscence with underlying, non‐adherent placenta: A systematic review of scoring systems and primary data analysis

**DOI:** 10.1111/aogs.14886

**Published:** 2024-05-31

**Authors:** Theophilus K. Adu‐Bredu, Rozi Aditya Aryananda, Joseph Arkorful, Sam Matthewlynn, Sally L. Collins

**Affiliations:** ^1^ Nuffield Department of Women's and Reproductive Health University of Oxford Oxford UK; ^2^ Obstetrics and Gynecology Department, Maternal Fetal Medicine, Dr Soetomo Academic General Hospital Universitas Airlangga Surabaya Indonesia; ^3^ Department of Medical Imaging University of Cape Coast Cape Coast Ghana

**Keywords:** bladder injury, cesarean hysterectomy, morbidly adherent placenta, PAS disorders, placenta accreta, placenta previa, scoring system

## Abstract

**Introduction:**

Accurate discrimination between placenta accreta spectrum (PAS) and scar dehiscence with underlying non‐adherent placenta is challenging both on prenatal ultrasound and intraoperatively. This can lead to overdiagnosis of PAS and unnecessarily aggressive management of scar dehiscence which increases the risk of morbidity. Several scoring systems have been published which combine clinical and ultrasound information to help diagnose PAS in women at high risk. This research aims to provide insights into the reliability and utility of existing accreta scoring systems in differentiating these two closely related but different conditions to contribute to improved clinical decision making and patient outcomes.

**Material and Methods:**

A literature search was performed in four electronic databases. The references of relevant articles were also assessed. The articles were then evaluated according to the predefined inclusion criteria. Primary data for testing each scoring system were obtained retrospectively from two hospitals with specialized PAS services. Each scoring system was used to evaluate the predicted outcome of each case.

**Results:**

The literature review yielded 15 articles. Of these, eight did not have a clearly described diagnostic criteria for accreta, hence were excluded. Of the remaining seven studies, one was excluded due to unorthodox diagnostic criteria and two were excluded as they differed from the other systems hindering comparison. Four scoring systems were therefore tested with the primary data. All the scoring systems demonstrated higher scores for high‐grade PAS compared to scar dehiscence (*p* < 0.001) with an excellent Area Under the receiver operator characteristic Curve ranging from 0.82 (95% CI 0.71–0.92) to 0.87 (95% CI 0.79–0.96) in differentiating between these two conditions. However, no statistically significant differences were noted between the low‐grade PAS and scar dehiscence on all scoring systems.

**Conclusions:**

Most published scoring systems have no clearly defined diagnostic criteria. Scoring systems can differentiate between scar dehiscence with underlying non‐adherent placenta from high‐grade PAS with excellent diagnostic accuracy, but not for low‐grade PAS. Hence, relying solely on these scoring systems may lead to errors in estimating the risk or extent of the condition which hinders preoperative planning.

## Graphical abstract

Most published studies on placenta accreta spectrum (PAS) scoring systems do not have a clearly defined diagnostic criteria. PAS scoring systems could differentiate between simple scar dehiscence from high‐grade PAS with high accuracy, but not low‐grade PAS.

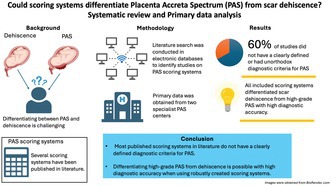

**Abbreviations**
CD: cesarean delivery
FIGO: International Federation of Gynecology and Obstetrics
PAS: placenta accreta spectrum
ULS: uterine lower segment

**Key message**

Placenta accreta spectrum (PAS) scoring systems accurately differentiate between scar dehiscence with underlying non‐adherent placenta from high‐grade PAS but not low‐grade PAS.

## 1 INTRODUCTION

Scar dehiscence with an underlying non‐adherent placenta is frequently misdiagnosed as placenta accreta spectrum (PAS) both prenatally and intraoperatively.^1^ This misinterpretation arises from the visualization of the placenta directly underneath a thinned uterine lower segment (ULS), a consequence of inadequate healing of the previous cesarean delivery (CD) scar.^1^, ^2^ This poor uterine healing leads to the extensive remodeling and architectural disruption of the ULS as the pregnancy advances, which manifests as placental bulge and myometrial thinning in scar dehiscence and high‐grade PAS both intraoperatively and on prenatal ultrasound.^1^, ^3^ Undoubtedly, PAS is an obstetrician's nightmare due to the risk of massive hemorrhage and severe morbidity. In suspected cases, there is a notable risk of confirmation bias among surgeons, especially when prenatal ultrasound inaccurately indicates PAS in instances of scar dehiscence and when the placenta appears beneath extensively remodeled ULS at laparotomy. This misinterpretation persists even when evaluated by seasoned experts in the field.^2^ Despite the promising research findings of advanced uterine sparing approaches for PAS in recent years,^4^, ^5^, ^6^ these advanced procedures are currently performed in only a few PAS specialist centers around the world where surgeons possess specialist skills in several pelvic devascularization and hemostasis control techniques. Hence, hysterectomy remains the management approach of choice for most centers globally, including well‐resourced hospitals in high‐income countries.^7^ In view of this, the misconception of dehiscence as PAS may trigger PAS‐appropriate management strategies like hysterectomy. While there is a growing body of evidence in recent years, supporting the significance of a standardized intraoperative PAS topographic staging system before implementing any management approach,^7^, ^8^, ^9^ prenatal diagnosis remains paramount for ensuring a comprehensive preoperative preparation.^10^, ^11^

As implied by its name, PAS is not a binary condition, it is a spectrum disorder which encompasses varying degrees of abnormal placental adherence as well as abnormal migration of extravillous trophoblast to deep planes of the uterine wall followed by uteroplacental remodeling.^10^ It is notable that varying degrees of PAS can occur in the same placental bed.^12^ This highlights the complexity and variability of PAS and explains the differences sometimes seen in prenatal imaging, clinical findings, and histopathological reports.^3^ Since 2008, numerous scoring systems have emerged in literature for identifying patients at risk of PAS. Each of these systems incorporates distinct clinical and ultrasound criteria in their algorithm with the aim of improving the predictive ability for identifying PAS.^12^, ^13^ Since then, several validation studies of the scoring systems have been published^14^, ^15^, ^16^, ^17^, ^18^ and are currently being used in some clinical settings around the world in assessing individuals at risk of PAS.^16^, ^19^, ^20^

Given the current diagnostic challenge in distinguishing between scar dehiscence and PAS, it is imperative to assess the efficacy of existing scoring systems in this regard. This study undertook a comprehensive literature review to identify all robustly developed published scoring systems and examined their effectiveness in differentiating between scar dehiscence and PAS using primary data analysis.

## 2 MATERIAL AND METHODS

### 2.1 Systematic review

#### 2.1.1 Eligibility criteria

All published primary studies that established scoring systems for the use of ultrasound in predicting PAS were eligible for inclusion. Studies that involved the use of scoring systems in predicting morbidity (blood loss, visceral organ injury) were excluded. Validation studies of the already published scoring systems were excluded. Also, articles that did not have a clearly defined diagnostic criteria for PAS (either intraoperative or histological) were excluded.

#### 2.1.2 Information sources and search strategy

The literature search encompassed PubMed, Scopus, Web of Science, and Google Scholar, capturing all relevant articles on the topic published up until August 22, 2023. The references of the relevant articles obtained were carefully screened to identify articles not captured in the electronic search. The search strategy was done by breaking down the research topic into the Population, Intervention, Comparator, Outcome (PICO) format. The Medical Subject Headings (MeSH) terms “Placenta Accreta,” “Ultraso*,” and “sonog” were combined with the other PICO terms and their synonyms. The search term used was (scor* OR index* OR model*) AND (accreta OR 'morbidly adherent' OR 'abnormally invasive' OR AIP OR 'pernicious') AND (placenta*) AND (ultras* OR sonog*).

#### 2.1.3 Study selection

Citations derived from the electronic databases were exported to Endnote version 21 citation manager (Carafate, Philadelphia). After removal of duplicates, the citations were then exported to Rayyan^20^ for independent screening and selection of eligible studies by TAB and JA. Initially, the title and abstracts were reviewed, followed by a thorough examination of the full text of the remaining articles to exclude those that did not meet the inclusion criteria.

#### 2.1.4 Data extraction and risk of bias analysis

**FIGURE 1:**
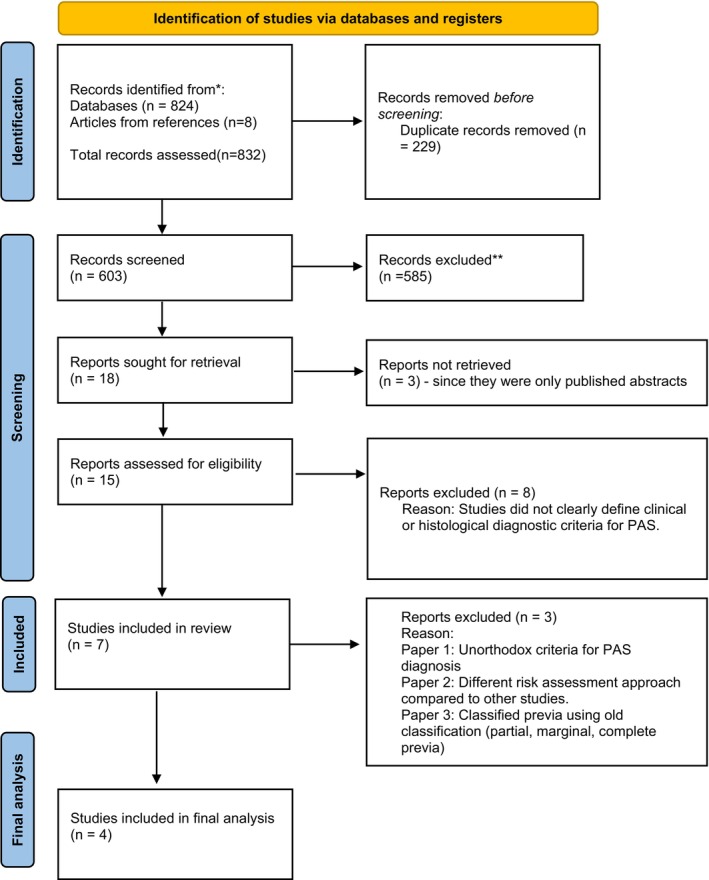
The PRISMA flow diagram.

The following data were extracted from the studies; Name of author, publication year, type of data used (retrospective or prospective), sample size, and ultrasound parameters. Subsequently, SC and TAB conducted a meticulous examination of the methodologies employed by the studies, independently assessing their diagnostic criteria for PAS. Those studies lacking clear descriptions or defined diagnostic criteria for PAS were excluded. The decision to exclude the articles based on diagnostic criteria was reached through consensus between the two reviewers. Figure 1 shows the PRISMA flow diagram.

### 2.2 Scoring system primary data analysis

#### 2.2.1 Materials and methods

Data were collected from two hospitals with specialized PAS services and expertise in management. Data for this retrospective analysis were obtained from the John Radcliffe Hospital, Oxford, UK (January 2019–December 2022) and Dr Soetomo Academic General Hospital, Surabaya, Indonesia (July 2022–October 2023). The study included all cases within the study period with low lying placenta and a previous CD referred to the placenta clinic. All cases where the placenta migrated to the anterior high position after the index scan were excluded. These cases were transferred for regular care and were delivered following routine protocol.

#### 2.2.2 Data acquisition and eligibility criteria

The ultrasound examinations were performed by RA and SC with extensive experience and expertise in PAS imaging. This was performed and reported with reference to the standard imaging descriptors for PAS imaging.^21^ Ultrasound examinations were performed with a full urinary bladder using transabdominal or transvaginal approach or a combination of both. All cases with more than two ultrasound imaging signs were considered as a high risk of PAS at the time of ultrasound.

In order for the data to be incorporated into the study, it was required to meet all the following criteria; clearly described and contemporaneously reported ultrasound findings, good quality ultrasound images and videos to compliment ultrasound reports (to enable case discussion), detailed description of the intraoperative findings (with or without pictures) and/or clearly described histological findings of the PAS cases. Cases that did not meet these inclusion criteria or had incomplete clinical data were excluded from the study.

The data from all cases meeting the inclusion criteria were reviewed by a second operator (TAB), then tabulated and transferred to a pre‐programmed Microsoft Excel data collection sheet equipped with the scoring systems. All discrepancies encountered were resolved by discussion with SC or RA. The scoring of cases for each scoring systems was done computationally, hence ensuring blinding to clinical outcomes, and mitigating the risk of transcription or arithmetic errors. Synopsis of the scores have been detailed in the Table S2

#### 2.2.3 Diagnostic criteria and clinical grading

Clinical diagnosis of PAS was made when the placenta was observed to be abnormally adherent to the uterine wall after delivering the baby. PAS was categorized using the International Federation of Gynecology and Obstetric (FIGO) clinical classification.^22^ For the purpose of this study, the PAS cases were classified based on intraoperative findings. Low‐grade PAS refers to the normal appearance of the ULS with mild or no vascularity on the serosal surface (FIGO Grade 1). High‐grade PAS was defined as the presence of a bulge, a thinned ULS with or without direct visualization of the placenta underneath, with hypervascularity on the serosa surface (FIGO Grades 2 & 3).

**FIGURE 2:**
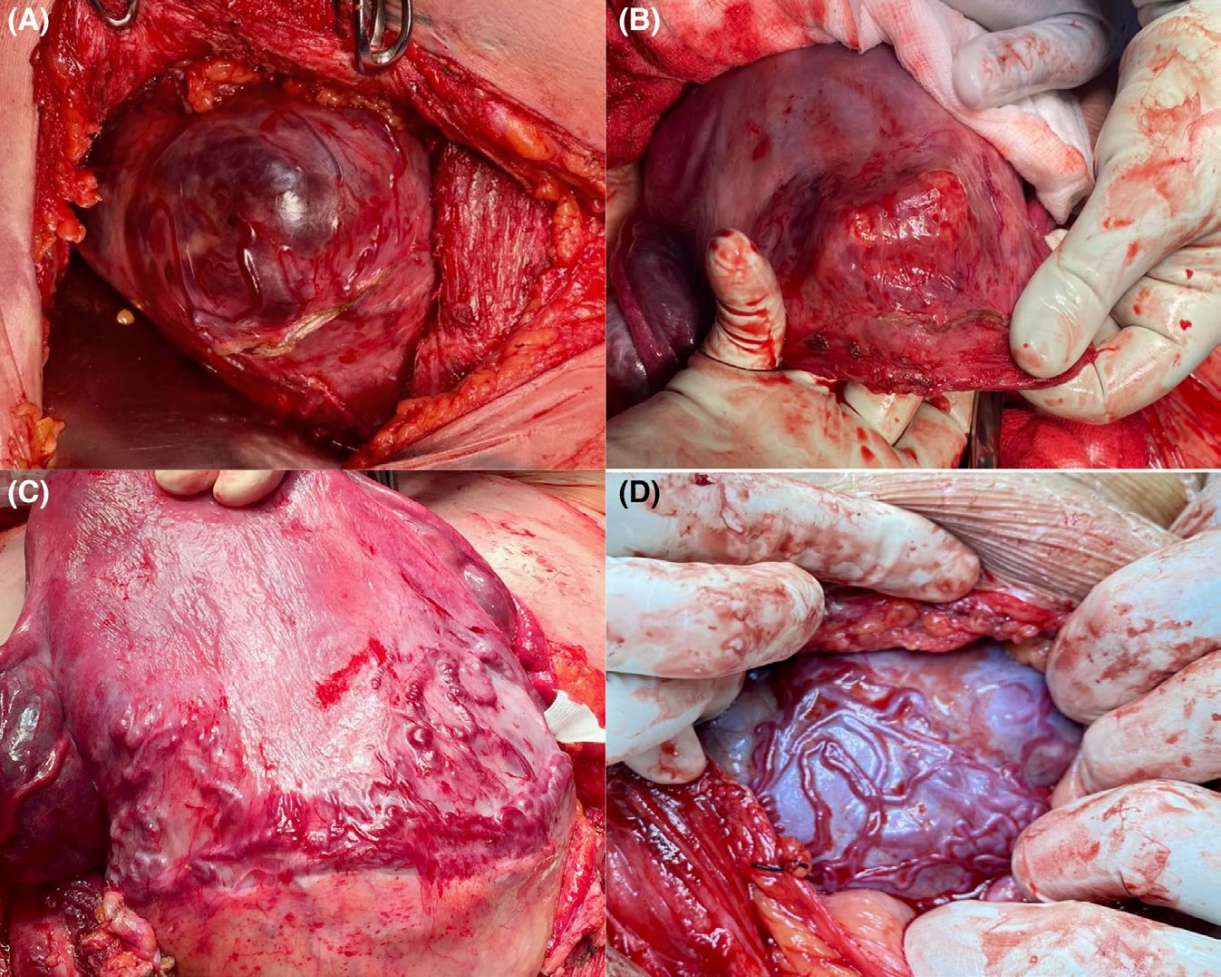
(A) Shows a case of scar dehiscence with non‐adherent placenta underneath, note the clearly circular shape of the uterine defect with the placenta directly visible underneath. The surrounding myometrium is completely normal. There are a couple of relatively straight vessels lying underneath the serosal surface (the appearance is that of them being draped in cling film) which arise at the edge of the defect, travel across the placental bed and then penetrate the underlying placenta. (B) Shows the surgeon's fingers in the posterior of the residual defect from the same case after the placenta separated with gentle controlled cord traction (the incision was placed under the defect to avoid cutting through the placenta given the difficulty in diagnosing dehiscence). There was minimal bleeding from the defect. (C and D) Show the neovasculaturity associated with high‐grade PAS. This is usually a web of tortuous vessels not contained within a regularly shaped defect (circular or elliptical) as seen with dehiscence, they appear to be lying within or on top of the serosa rather than being draped with it.

Scar dehiscence with a non‐adherent placenta underneath was defined as the presence of the placenta under a thin, transparent lower segment with an obvious bulge, visible placenta with notable absence of neovascularity on the serosa surface and normal surrounding uterine tissue with detachment of the placenta from the uterus surrounding the defect^12^ (Figure 2).

Uncomplicated placenta previa was defined as the presence of a normal looking ULS at laparotomy with complete placenta separation after delivering the fetus.

Histological diagnosis was also based on the FIGO classification^22^; Accreta was defined as the direct attachment of the placental villi to the myometrium; Increta was defined as the presence of placenta villi within myometrial tissue; and Percreta was defined as the presence of the placenta villi within or extending beyond the uterine serosa.

#### 2.2.4 Management

The management approach for PAS was based on an individualized approach, considering the preference of the woman and safety. In one center, cesarean hysterectomy or intentional placental retention was offered to the woman. In the cases that underwent intentional placenta retention, histological analysis was not possible, and the diagnosis of PAS relied on the FIGO Clinical Classification. The cases were managed by an experienced multidisciplinary team led by an obstetrician and gyne‐oncologist highly experienced in PAS.

For the second center, the women were offered the options of cesarean hysterectomy or the possibility of one‐step conservative surgery.^4^ In those who opted for one‐step conservative surgery, the final decision on the management approach whether one‐step conservative surgery, cesarean hysterectomy or modified subtotal hysterectomy was made intraoperatively, based on the placental topographic classification system.^7^ All cases were managed by RA, an experienced obstetrician in PAS surgery.

In the cases where scar dehiscence was present with a non‐adherent placenta underneath, the management approach was placenta removal either spontaneously or with controlled cord traction followed by uterine reconstruction in one center. In the second center, it was managed using the One‐step conservative surgery technique.

#### 2.2.5 Statistical analyses

Data were collected in Microsoft Excel (Office 365; Microsoft corporation, Redmond, Washington, USA) and exported to IBM SPSS statistical software package (IBM, Chicago, Illinois, USA) version 29 for analysis. Categorical variables were described as frequencies with corresponding percentages presented in tables. Continuous or numerical variables were expressed as median with interquartile values. The Kruskal–Wallis test was used to analyze the differences among group means. Afterwards, post hoc analysis was performed to determine pairwise comparisons. Corrections for multiple comparisons were not applied. Diagnostic tests of accuracy of the different scoring systems were determined using receiver operator characteristic (ROC) curves and a cut‐off value for each scoring system was obtained from the results. A 95% confidence interval with a *p* value of 0.05 was used to determine statistical significance.

## 3 RESULTS

### 3.1 Systematic review

#### 3.1.1 Literature search and eligibility criteria

The electronic database search yielded 824 results with the following breakdown: PubMed, 500, Scopus, 30, Web of Science, 202, and Google scholar, 92. Eight additional articles were retrieved from the references of relevant articles. Finally, 832 records were evaluated. After removal of duplicates and screening of the articles according to the inclusion criteria, 15 articles remained.^19^, ^24^, ^25^, ^26^, ^27^, ^28^, ^29^, ^30^, ^31^, ^32^, ^33^, ^34^, ^35^, ^36^, ^37^ Upon evaluating the diagnostic criteria for PAS in the articles related to the PAS scoring systems, only seven explicitly outlined the intraoperative and/or histological diagnostic criteria (Table S1). Of the seven articles, one was excluded due to unorthodox diagnostic criteria for PAS.^29^ Another was excluded^23^ since the algorithms for predicting the probability of PAS were binary, rather than score‐based, which hindered direct comparison with other studies. Another study^19^ was excluded because its scoring system graded placenta previa using the old classification (Partial, marginal, complete previa) which is not consistent with current clinical standards.^37^, ^38^ Therefore, it could not be applied to our data.

#### 3.1.2 Study characteristics

Four studies were included in our analysis.^25^, ^26^, ^31^, ^32^ Two of these employed a prospective study design, while the remaining two used a retrospective approach. The number of PAS cases used to develop the scoring system ranged from 23 to 54. The total number of PAS cases included in the studies was 144. The sonographic signs assessed in all the four studies were either derived from, or were variations of, the standardized ultrasound signs.^21^ The ultrasound examinations were performed using transabdominal and/or transvaginal ultrasound in the third trimester of pregnancy in all the studies, with exception of Tovbin et al.^32^ that performed some ultrasound examinations in the second trimester. Details of the studies have been highlighted in Table 1.

**TABLE 1:** Characteristics of included studies.

Study	Study design	Inclusion criteria	Sample size		Scanning approach	Trimester
			PAS	Non‐PAS		
Rac et al. (2015)	Retrospective	≥1 previous CD with low‐lying placenta or previa	54	130	Transabdominal and Transvaginal	3rd trimester
Tovbin et al. (2016)	Prospective	At least one of the following; Previous CD, myomectomy, history of PAS, ultrasound suspicion of PAS	23	235	Transabdominal and/or Transvaginal	2nd or 3rd trimester
Del Negro et al. (2020)	Retrospective	≥1 previous CD with low‐lying or placenta previa	29	109	Transabdominal and Transvaginal	3rd trimester
El‐Haieg et al. (2019)	Prospective	≥1 previous CD with low‐lying or placenta previa	38	76	Transabdominal and Transvaginal	3rd trimester

Abbreviations: CD, cesarean delivery; PAS, placenta accreta spectrum.

#### 3.1.3 Risk factors

Currently, the primary cause of PAS is the presence of placenta underlying the previous CD scar. All scoring systems considered incorporated this combination into their models, except for El‐Haieg et al.^26^ In the study by El‐Haieg et al.^26^ this risk factor was not included in their scoring model. While Tovbin et al.^32^ and Del Negro et al.^25^ assigned higher scores for an increasing number of previous CDs, Rac et al.^31^ and Del Negro et al.^25^ did not allocate scores to patients with only one previous CD. Except for the number of previous CDs, none of the studies included any other known risk factors of PAS into their algorithms.

#### 3.1.4 Ultrasound signs

Features of abnormal ULS remodeling have been characterized to be myometrial thinning, placental bulge, and loss of retroplacental clear zone. All the studies included one or more features of ULS remodeling in their scoring system. The presence of placenta bulge was only assessed by Del Negro et al.^25^ The retroplacental clear zone^25^, ^26^, ^32^ and myometrial thinning^25^, ^26^, ^31^ were assessed by three studies out of the four included studies.

All studies evaluated features of abnormal vascularity. The presence of lacunae was graded in each study, with higher scores assigned for increasing numbers in all the studies.^25^, ^26^, ^31^, ^32^ Likewise, hypervascularity (subplacental/uterovesical) were evaluated, with two studies^25^, ^26^ assigning higher scores based on increasing color Doppler signal intensity, while one study^32^ considered them as a binary variable. Rac et al.^31^ however, did not assess or integrate features of subplacental or uterovesical hypervascularity into the model, instead, provided a score for the presence of bridging vessels.

#### 3.1.5 Primary data analysis

The study assessed a total of 150 cases of low‐lying placenta or previa with at least one previous CD. However, only 144 met the inclusion criteria and so were used to assess the scoring systems. These cases comprised 89 cases of PAS involving 16 cases of low‐grade PAS and 73 cases of high‐grade PAS. The remaining 55 cases of non‐PAS cases comprised of 32 cases of uncomplicated previa and 23 cases of scar dehiscence. The median gestation age at ultrasound was 34 weeks (interquartile range 32–36) and the median gestational age at delivery was 36 weeks (interquartile range 34–37). Comprehensive details of the patient demographics, clinical and histological diagnosis as well as the management approach are presented in Table 2.

**TABLE 2:** Demographics, diagnosis, management, and outcomes of patients within the cohort.

Variables	Median (IQR) or *n* (%)
Demographics	
Number of cases	144
Maternal age(years)	34 (31–37)
Parity	
1	57 (40%)
≥2	87 (60%)
Number of previous CD	
1	77 (54%)
2	53 (37%)
≥3	14 (10%)
Number of STOPs	
None	105 (73%)
1	28 (19%)
≥2	11 (8%)
Gestational age at ultrasound (weeks)	34 (32–36)
Gestational age at delivery(weeks)	36 (34–37)
Diagnosis	
PAS	
Low‐grade PAS	16 (11%)
High‐grade PAS	73 (51%)
Non‐PAS	
Scar dehiscence	23 (16%)
Uncomplicated previa	32 (22%)
Management approach	
Uterine sparing surgery	96 (67%)
Cesarean hysterectomy	45 (31%)
Intentional placental retention	3 (2%)
Outcomes	
Blood loss (mL)	1500 (900–3000)
Composite maternal morbidity	15 (10%)
Histological classification (number of PAS cases = 89)	
Accreta	16 (18%)
Increta	60 (67%)
Percreta	11 (12%)
Histology unavailable	2 (2%)

Abbreviation: CD, cesarean delivery.

Analysis was done using the Kruskal‐Wallis test to compare the scores between the subgroups of the PAS (low grade and high grade) and non‐PAS cases (scar dehiscence and uncomplicated previa) and revealed a statistically significant difference among all the different PAS scoring systems (*p* < 0.001). Post hoc pairwise comparisons found no statistically significant difference between the scores obtained for scar dehiscence and low‐grade PAS in all the four different scoring systems. In contrast, the scores obtained for high‐grade PAS were significantly higher than scar dehiscence among all the scoring systems (*p* < 0.001; Table 3). Diagnostic test of accuracy revealed an Area Under the Receiver operator characteristic Curve (AUC) ranging from 0.82 (95% CI 0.71–0.92) to 0.87 (0.79–0.96) for all the different scoring systems in differentiating scar dehiscence and high‐grade PAS (Table 4).

**TABLE 3:** Comparative analysis of the scores between different groups.

Scoring systems	Main groups			PAS subgroups		Non‐PAS subgroups		*p* values			
	PAS	Non‐PAS	*p* value	Low‐grade PAS	High‐grade PAS	Dehiscence	Uncomplicated previa	High grade vs. low grade	High grade vs. dehiscence	Low grade vs. dehiscence	Dehiscence vs. uncomplicated previa
Rac et al.	7 (2–10)	3 (1.25–8.5)	<0.001	6.5 (2.25–10)	7 (2–10)	5 (2–8.5)	1.25 (1.25–4.25)	<0.001	<0.001	0.121	0.002
Del Negro et al.	16 (7–21)	6 (2–17)	<0.001	7 (5–11)	17 (10–21)	11 (8–17)	4 (2–6)	<0.001	<0.001	0.135	<0.001
Tovbin et al.	11 (4–12)	6 (3–11)	<0.001	9 (5–12)	11 (4–12)	5 (3–11)	6 (4–8)	<0.001	<0.001	0.084	0.485
El‐Heig et al.	9 (2.5–9.5)	0.5 (0–9.5)	<0.001	7.5 (2.5–9)	9 (5.5–9.5)	6.5 (5.5–9.5)	0 (0–4)	<0.001	<0.001	0.662	<0.001

*Note*: Median (range).

**TABLE 4:** Shows the diagnostic test of accuracy results of each scoring system in differentiating high‐grade PAS from scar dehiscence with underlying non‐adherent placenta.

Scoring systems	Cut‐off	AUC (95% CI)	Sensitivity (95% CI) %	Specificity (95% CI) %
Rac score	6.0	0.86 (0.78–0.94)	83.6 (73.1–91.2)	82.6 (61.2–95.1)
Del Negro score	15.5	0.87 (0.79–0.96)	78.1 (66.9–86.9)	82.6 (61.2–95.1)
Tovbin score	5.5	0.82 (0.71–0.92)	98.6 (92.6–99.97)	52.2 (30.6–73.2)
El‐Heig score	8.25	0.83 (0.73–0.94)	71.2 (59.5–81.2)	82.6 (61.2–95.1)

With exception of the scoring systems derived by Tovbin et al.^32^ that did not show statistically significant differences between scar dehiscence and uncomplicated placenta previa (*p* = 0.485), all the other scoring systems showed significant differences between the scar dehiscence and uncomplicated previa (Table 3; Figure 2).

## 4 DISCUSSION

Results from our systematic review revealed that 8 of the 15 published studies on PAS scoring systems did not clearly describe the intraoperative or histological diagnosis of PAS. Instead, they primarily relied on the opinion of the surgeon at delivery with no detailed description of how this was reached. One study defined the various grades of PAS unconventionally, as they defined Accreta as manual placenta removal with residual placental diameter less than 1 cm and increta/percreta as placenta removal requiring scissors.^29^ Similarly, meta‐analysis on the epidemiology of PAS showed significant heterogeneity in the diagnostic criteria of PAS.^39^ Generally, in medical practice and research, the diagnostic criterion for a condition is the key in understanding the epidemiology, etiology, and management approach which influences guidelines for practice. However, the abundance of published studies with unclear diagnostic criteria, increases the risk of bias and brings the authenticity of the published data on PAS into question. Indeed, the trajectory of PAS has transitioned from being significantly underdiagnosed in the past decade to probable overdiagnosis now. As evidenced in our systematic review, the highest number of PAS cases recorded were of those with doubtful or unclear diagnostic criteria. The diagnostic criteria, management, and outcomes of this rare condition vary strikingly between hospitals and are often based on opinion. This unfortunately impedes a comprehensive evaluation of data crucial for informing guidelines for medical practice and the training of the next generation of obstetric practitioners, ultimately impacting patient safety.

The publication of numerous PAS scoring systems has led to scrutiny among some experts due to the incorporation of clinical and ultrasound information that are common with scar dehiscence. Also, the co‐existing combination of scar dehiscence and PAS in the same placental bed in many cases makes prenatal diagnosis even more challenging. The crucial inquiry for the surgical or management team is “What can we anticipate?” which facilitates appropriate case assignment and ensures thorough preparation. One may argue that PAS and scar dehiscence with underlying placenta all carry morbidity risk and accurate prenatal differentiation may not be relevant.^40^ Even though we agree that a very large dehiscence may occasionally need a hysterectomy, the degree of placental attachment, management approach, resource allocation, and degree of morbidity are significantly different from PAS. The morbidity associated with PAS in previa usually results from the inability to secure hemostasis due to the neovascularity and anomalous blood supply to the uterus that occurs, the surgical risk of transecting the placental bed and the collateral damage to surrounding organs like the ureter and bladder.^10^ In contrast, the management and morbidity associated with scar dehiscence is dependent on the availability of healthy myometrial tissue above the cervix for uterine reconstruction. Our study revealed that the scoring systems differentiated between scar dehiscence with a non‐adherent placenta underneath and high‐grade PAS with an excellent diagnostic accuracy (Table 3). However, the scoring systems were inadequate in differentiating between the low‐grade PAS and scar dehiscence (Table 2). These results hold relevance, since the intraoperative diagnostic dilemma has been between high‐grade PAS and scar dehiscence. Studies evaluating the uteroplacental and vesicouterine interfaces in the cases of scar dehiscence showed the absence of abnormal vascularity which is typically seen in high‐grade PAS.^2^, ^3^, ^42^ When analyzing the scoring systems, the individual features of abnormal vascularity which included uteroplacental vascular remodeling (abnormal lacunae, uteroplacental, or uterovesical hypervascularity) and serosal hypervascularity (bladder wall interruption and bridging vessels) were integrated in the model which explains the lower scores observed in scar dehiscence compared to high‐grade PAS (Table 2). In contrast, low‐grade PAS lacks the overt signs of abnormal vascularity^22^ which may explain the similar scores observed with scar dehiscence.

Despite the absence of abnormal vascular changes in scar dehiscence,^41^ we would like to emphasize that the placenta by nature is a vascular organ; hence, normal subplacental vascularity could be misdiagnosed as PAS particularly in the presence of significant risk factors for PAS. Also, urinary bladder varices which is a common finding in pregnancy can be easily mistaken for uterovesical hypervascularity.^42^, ^43^ Placenta lakes and infarcts could be mistaken for abnormal lacunae.^10^, ^44^, ^45^ It is therefore worth emphasizing that improper utilization of these scoring systems could lead to high false positive and negative implications, potentially misleading the management team. Therefore, one should exercise caution and be mindful of all sonographic pitfalls when screening high‐risk women.

The strength of this study lies in the rigorous nature of literature search and evaluation of selected articles for the diagnostic criteria of PAS. This ensured that all relevant articles on the topic were obtained and properly evaluated. Furthermore, the scoring system analysis was done computationally which reduces the risk of bias and the potential for arithmetic error. A limitation of the study is the reliance on retrospective data to determine the efficiency of these scoring systems. Even though we acknowledge that this study would have benefited from a prospective study, our multicenter data from PAS specialist centers and rigorous inclusion criteria of the cases for analysis is a notable strength. Also, since ultrasound data was discussed between authors and finalized prior to computing the scores under each scoring system, our methodology did not allow for the determination of inter‐observer reliability.

## 5 CONCLUSION

Most sonographic scoring systems for predicting PAS published in literature do not have a rigorous and clearly defined intraoperative and/or histological diagnostic criteria for PAS. The included PAS scoring systems could differentiate between scar dehiscence with underlying non‐adherent placenta from high‐grade PAS with an excellent diagnostic accuracy. However, their ability to differentiate scar dehiscence from low‐grade PAS was limited. Hence, solely relying on these scoring systems may lead to errors in estimating the risk or extent of the condition which hinders a comprehensive preoperative planning. We therefore recommend that these scoring systems should be used as complementary tools rather than substitutes for intraoperative evaluation.

## AUTHOR CONTRIBUTIONS

Theophilus K. Adu‐Bredu and Sally L. Collins conceived and planned the methodology, Theophilus K. Adu‐Bredu and Joseph Arkorful performed the literature search; Theophilus K. Adu‐Bredu and Sally L. Collins scrutinized the articles for the inclusion criteria, Theophilus K. Adu‐Bredu, Rozi Aditya Aryananda and Sam Matthewlynn planned and contributed to the statistical analysis, Theophilus K. Adu‐Bredu drafted the original manuscript. Sally L. Collins supervised the study and reviewed the methodology. All authors contributed equally to finalizing the manuscript.

## CONFLICT OF INTEREST STATEMENT

All authors declare no conflict of interest.

## ETHICS STATEMENT

Ethics approval was obtained from these two institutions prior to data collection and analysis for this study. For John Radcliffe Hospital, ethics approval with reference number 14/NS/0069 was issued on May 22, 2014. For Dr Soetomo Academic General Hospital, ethics approval was obtained on December 19, 2022, with reference number 1169/LOE/301.4.2/XII/2022.

## Supporting information


Table S1.

Table S2.
