# Supplementary material for: Differentiating placenta accreta spectrum from scar dehiscence with underlying, non‐adherent placenta: A systematic review of scoring systems and primary data analysis
Source: Acta Obstet Gynecol Scand. 2024 May 31;104(Suppl 1):45–55. doi: 10.1111/aogs.14886 (PMC12087408; doi:10.1111/aogs.14886)
Supplement: Supplementary file 1 — Table S1. [file AOGS-104-45-s002.docx]

Supplementary table 1: evaluating the diagnostic criteria used in the articles.

| **Articles** | **Study design** | **Number of PAS cases** | **Diagnostic criteria** | | | **Included?** | **Reason, if not included** |
| --- | --- | --- | --- | --- | --- | --- | --- |
|  |  |  | Clearly defined Histology criteria | clearly defined intraoperative grading | Criteria used |  |  |
| Aiob, 2023^24^ | Retrospective | 59 | Yes | Yes | FIGO criteria^23^ | Yes | N/A |
| Chong, 2018^25^ | Prospective | 137 | Unclear | Not clearly defined | Clinical opinion  Histology was performed on an undefined number of cases | No | Diagnosis of PAS was based on an undefined clinical criterion. Also, histological confirmation was performed for an undisclosed number of people which increases the risk of bias |
| Del Negro, 2020^26^ | Retrospective | 29 | Yes | Yes | FIGO criteria^23^ | Yes | N/A |
| El – Haieg, 2019^27^ | Prospective | 38 | Yes | No | Histology examination based on a predefined criteria | Yes | N/A |
| Gao, 2021^28^ | Retrospective | 90 | No | No | Clinical opinion | No | Diagnosis was based entirely on clinical opinion in patient records which increases the risk of bias. |
| Gilboa, 2015^29^ | Retrospective | 76 | Unclear | No | Clinical opinion  Histology was performed on an undefined number of cases | No | Diagnosis of PAS was based on an undefined clinical criterion. Also, information on the cases that had histology was not reported. This increases the risk of bias. |
| Luo, 2019^30^ | Prospective | 160 | Yes  Unclear (to be discussed) | Yes  (unorthodox) | Accreta was defined clinically based on the residual placenta diameter <1cm. If removal required scissors, this was defined as increta or percreta. A further criterion of myometrial invasion being increta and percreta, beyond the serosa. | No | Unorthodox definition and diagnostic criteria for PAS. This increases the risk of bias. |
| Marsoosi, 2020^31^ | Prospective | 49 | Unclear | Unclear | Clinical opinion based on massive bleeding, difficulty to separate placenta or partial placenta separation.  Pathology results but undefined number of cases and criteria used | No | Clinical diagnosis involved cases with placenta bed bleeding after placental separation which may not be PAS particularly in the presence of previa. Also, the criteria of histological analysis and results from histology were not reported. This increases the risk of bias. |
| Rac, 2015^32^ | Retrospective | 54 | Yes | No | Histology based on the depth of invasion | Yes | N/A |
| Tovbin, 2016^33^ | Prospective | 23 | Yes | Yes | Clinical opinion – Based on inability to separate placenta.  Histology based on the depth of invasion | Yes | N/A |
| Wong, 2008^34^ | Retrospective and Prospective. | 9 | No | No | Histology with unclear criteria | No | Unclear criteria used in PAS diagnosis |
| Yang, 2022^19^ | Retrospective | 105 | Yes | Yes | FIGO criteria^23^ | Yes | N/A |
| Zhang, 2023^35^ | Prospective | 348 | No | No | Clinical opinion | No | Undefined clinical criterion used by the obstetricians for PAS diagnosis which increases the risk of bias. |
| Zheng, 2022^36^ | Retrospective | 2219 | No | No | Clinical opinion | No | Undefined clinical criterion used by the obstetricians for PAS diagnosis which increases the risk of bias. |
| Zhu, 2019^37^ | Prospective | 36 | unclear | unclear | Clinical opinion  Histology was performed on an undefined number of cases | No | Diagnosis of PAS was based on an undefined clinical criterion. Also, histological confirmation was performed for an undisclosed number of people with results unreported which increases the risk of bias |
