# Supplementary material for: Differentiating placenta accreta spectrum from scar dehiscence with underlying, non‐adherent placenta: A systematic review of scoring systems and primary data analysis
Source: Acta Obstet Gynecol Scand. 2024 May 31;104(Suppl 1):45–55. doi: 10.1111/aogs.14886 (PMC12087408; doi:10.1111/aogs.14886)
Supplement: Supplementary file 2 — Table S2. [file AOGS-104-45-s001.docx]

| **Scoring system** | **Parameters and scores** | | | **Value** |
| --- | --- | --- | --- | --- |
| Rac et al(32) | > 2 caesarean deliveries | | | 3.0 |
|  | Lacunae | Grade 3 | | 3.5 |
|  |  | Grade 2 | | 1.0 |
|  | Sagittal smallest myometrial thickness | < 1mm | | 1.0 |
|  |  | < 1 but > 3mm | | 0.5 |
|  |  | >3 but < 5mm | | 0.25 |
|  | Anterior placenta previa | | | 1.0 |
|  | Bridging vessels | | | 0.5 |
| Tovbin et al(33) | Number of previous caesarean deliveries | 1 | | 1 |
|  |  | > 2 | | 2 |
|  | Lacuna maximum dimension | < 2cm | | 1 |
|  |  | > 2cm | | 2 |
|  | Number of lacunae | < 2 | | 1 |
|  |  | > 2 | | 2 |
|  | Obliteration of uteroplacental demarcation | | | 2 |
|  | Location of placenta | Anterior | | 1 |
|  |  | Placenta previa | | 2 |
|  | Doppler assessment | Blood flow in placenta lacunae | | 1 |
|  |  | Hypervascularity (placenta-bladder and/or uteroplacental interface | | 2 |
| El- Haieg et al(27) | Retroplacental clear zone | | | 2 |
|  | Myometrial thickness < 1mm | | | 2.5 |
|  | Hyperechoic uterine -urinary bladder interface | | | 1 |
|  | Placenta lacunae | | >4, large size (> 1 x 1cm), irregular and/or diffuse | 2 |
|  | Uterine serosal-bladder wall interface and intraplacental and bladder wall vascularity by color Doppler | | Moderate | 0.5 |
|  |  |  | Increased | 1 |
| Del Negro et al(26) | Number of previous caesarean deliveries | | One | 0 |
|  |  |  | Two | 1 |
|  |  |  | Three or more | 2 |
|  | Position of placenta | | Low-lying | 1 |
|  |  |  | Previa | 2 |
|  | Placenta lacunae | | Finberg’s Grade 1 | 1 |
|  |  |  | Finberg’s Grade 2 or 3 | 2 |
|  | Retroplacental space | | Present | 0 |
|  |  |  | Irregular | 1 |
|  |  |  | Absent | 2 |
|  | Retroplacental myometrial thickness | | >1mm | 0 |
|  |  |  | < 1mm | 1 |
|  |  |  | Not measurable | 2 |
|  | Bladder wall | | Line clear and complete | 0 |
|  |  |  | Line vague or irregular | 1 |
|  |  |  | Line lost | 2 |
|  | Focal exophytic mass and/or placenta bulge | | Present | 2 |
|  |  |  | Absent | 0 |
|  | Uterovesical hypervascularity | | Normal | 0 |
|  |  |  | Increased flow, presence of numerous vases, tortuous | 1 |
|  |  |  | Multidirectional flow or presence of bridging vessels | 2 |
|  | Subplacental hypervascularity | | Normal | 0 |
|  |  |  | Increased flow | 1 |
|  |  |  | Bridging vessels with perpendicular course | 2 |
|  | Diffuse or focal turbulent flow in the lacunae | | Absent | 0 |
|  |  |  | Focal | 1 |
|  |  |  | Diffused with tributary vessels | 2 |

Table S2. Synopsis of all the parameters and corresponding scores of the studies included in our statistical analysis.
